# Effect of tanshinone IIA on cardiomyocyte hypertrophy and apoptosis in spontaneously hypertensive rats

**DOI:** 10.3892/etm.2013.1339

**Published:** 2013-10-10

**Authors:** F.L. JIANG, S. LEO, X.G. WANG, H. LI, L.Y. GONG, Y. KUANG, X.F. XU

**Affiliations:** Department of Cardiology, Third Xiangya Hospital, Central South University, Changsha, Hunan 410013, P.R. China

**Keywords:** left ventricular hypertrophy, apoptosis, tanshinone IIA, p53, Bcl-2, Bax

## Abstract

In the present study, the effects of tanshinone IIA (TSN) on the prevention of left ventricular hypertrophy (LVH) and apoptotic processes were observed in spontaneously hypertensive rats (SHRs). A total of 18 SHRs (age, 8 weeks) were randomly divided into three groups. The SHRs in the control group (group S8) were sacrificed at week 8 of the experiment. The SHRs in the treatment group (group D18) and the placebo group (group S18) were injected with TSN and distilled water (1 ml/kg body weight/day), respectively, for 10 weeks, commencing at week 8, and were subsequently sacrificed at week 18. The systolic blood pressure (SBP) and left ventricular mass index (LVMI) were determined. Using hematoxylin and eosin and van Gieson staining, together with immunohistological methods, cardiomyocyte size and diameter, collagen volume fraction (CVF) and perivascular circumferential area (PVCA) were measured. Evaluation of Bcl-2, Bax and p53 expression levels for apoptosis analysis was performed using western blotting. It was observed that the SBP, LVMI, cardiomyocyte size and diameter, CVF, PCVA and cardiomyocyte apoptosis index (Bax and p53 expression) were increased significantly in group S18 compared with group S8. However, Bcl-2 expression levels were decreased in group S18 compared with group S8. The administration of TSN in group D18 resulted in higher Bcl-2 expression levels and significantly decreased LVMI, cardiomyocyte size and diameter, CVF, PCVA, Bax and p53 expression levels compared with group S18. LVH and apoptosis of the cardiac tissues increased with the increasing age of the SHRs. TSN may inhibit the development of LVH and decrease the level of apoptosis in SHRs, possibly via the upregulation of Bcl-2 and the downregulation of Bax and p53 expression.

## Introduction

Left ventricular hypertrophy (LVH) is a commonly encountered complication of hypertension (1). It is also an independent risk factor for the development of cardiovascular disease, since it may lead to myocardial ischemia, cardiac arrhythmia, heart failure and even sudden death (2,3). Tanshinone IIA (TSN), the main active compound of the Traditional Chinese Medicine *Salvia miltiorrhiza*, has been known to exert therapeutic effects in heart disease via its vasodilative (4), atheroprotective (5), antiarrhythmic (6) and anti-inflammatory properties (7). In our previous study (8), the cardioprotective effects of TSN in preventing the development of LVH were demonstrated in spontaneously hypertensive rat (SHR) models. A likely mechanism for this may be that TSN inhibited the renin-angiotensin-aldosterone system (RAAS) (9). High RAAS activity in the presence of LVH has previously been associated with increased an increased level of apoptosis (10). Therefore, the present study investigated the role of TSN, extracted from *Salvia miltiorrhiza*, in the development of LVH and the apoptotic protein expression in SHRs.

## Materials and methods

### Animal model and grouping

All procedures were in accordance with the international, national and institutional rules on animal experiments. Legal approval from The Review Board of Xiangya Third Hospital of Central South University (Changsha, China) was granted for the experiments undertaken. A total of 18 male SHRs (Shanghai Institute of Hypertension, Shanghai, China) were used in this study. The rats were fed a standard rat diet and bred in a constant temperature and humidity for the first 8 weeks before applying any intervention. The rats were randomly divided into three groups with six SHRs in each group. Rats in the control group (group S8) were sacrificed at week 8 of the experiment. Rats in the treatment group (group D18) were injected with *Salvia miltiorrhiza* TSN (1 ml/kg body weight/day; S02001300; National Institute for the Control of Pharmaceutical and Biological Products, Beijing, China) for 10 weeks, commencing at week 8, and were subsequently sacrificed week 18. The placebo group (group S18) were treated similarly to group D18, although distilled water (1ml/kg body weight/day) was administered instead of TSN.

### Reagents

The main reagents used in this study were TSN (China Pharmaceutical and Biological Products Inspection Center) and rabbit anti-Bcl-2, Bax and p53 monoclonal antibodies (Santa Cruz Biotechnology, Inc., Shanghai, China). In addition, a terminal deoxynucleotidyltransferase-mediated dUTP nick end labeling (TUNEL) assay kit (Boehringer, Mannheim, Germany) was used.

### Measurements

#### Systolic blood pressure (SBP) measurement

Following warming the rats at 38ºC for 15 min, SBP was measured using a tail*-*cuff sphygmomanometer (MRS2 III, Shanghai Institute of Hypertension). Five measurements of systolic pressure were taken for each rat and the SBP was defined as the mean value of the five measurements. All procedures were performed with animals in a conscious state.

#### Left ventricular mass index (LVMI)

Following sacrifice, the hearts of the rats were removed. The major vessels, the atria and the right ventricle were separated from the left ventricle, prior to the left ventricle being washed with cold phosphate-buffered saline (PBS) and dried. The wet weight (mg) of the left ventricle was then measured. The LVMI was defined as the value of the left ventricular weight (mg) divided by the total body weight (g).

#### Qualitative and quantitative analysis of cardiac myocytes and collagen

Tissue samples were obtained for analysis from the transverse plane of the middle of the left ventricle. Following fixation, the tissue was embedded and sliced for hematoxylin and eosin (H&E) and van Gieson (VG) staining. The diameter and area of cardiac myocytes were measured under H&E staining using fully automatic morphometry equipment (HPIAS21000, Tongji Medical Imaging Engineering Co., Wuhan, China). Under VG staining, myocytes appeared yellow and collagen appeared red. The collagen volume fraction (CVF) of myocardial tissue and the perivascular circumferential area (PVCA) were measured under VG staining. The CVF was defined as the PVCA divided by the total area of the sample. The PVCA was defined as the collagen area surrounding the arteriole divided by the arterial lumen area. All values were calculated as the mean value obtained from five random measurements for each specimen.

#### Analysis of cardiac myocyte apoptosis

Cardiac myocyte apoptosis was analyzed using the TUNEL method. Following dewaxing, cardiac tissue was hydrated with alcohol and immersed in protease K (20 μg/ml; Sigma-Aldrich, Shanghai, China) at 37ºC for 15 min. Peroxidase (POD) activity was deactivated by the addition of hydrogen peroxide-carbinol (0.3%; Sigma-Aldrich). The sample was washed with PBS four times prior to 30 μl TUNEL mixture (Boehringer) being added, and the sample then was incubated in a wet box for 1 h at 37ºC. Subsequently, the tissue was re-washed with PBS, and re-incubated for 30 min. Following incubation, the tissue was washed with PBS and 3,3′-diaminobenzidine (DAB) revealed its color. The tissue was then stained with H&E. The nuclei of normal cells appeared blue while those of apoptotic cells appeared red.

#### Western blot assay of Bcl-2, Bax and p53 genes

TRIzol^®^ (Invitrogen Life Technologies, Carlsbad, CA, USA) was added to 50 mg myocardial tissue for protein extraction. Quantitative analysis of the protein was performed according to Bradford’s method. Protein and buffer were mixed in the ratio 1:3, boiled for three min prior to vertical electrophoresis in sodium dodecyl sulfate (SDS) polyacrylamide gel (separation gel 8% and layering gel 4.5%) and transferred to a polyvinylidene difluoride (PVDF) membrane. The membrane was blocked with TBS2T (with 5% skimmed milk powder) for 2 h at room temperature. Bcl-2, Bax and p53 monoclonal antibodies (Santa Cruz Biotechnology, Inc.; 1:500 dilution) were added to the samples and incubated at 4ºC for one night. The following day, membranes were washed four times with TBS2T, prior to horseradish peroxidase-conjugated goat anti-rabbit immunoglobulin G (IgG) antibodies (1:8,000 dilution; Sigma-Aldrich) being added. The samples were then agitated for 2 h and re-washed with TBS2T four times. Subsequently, enhanced chemiluminescence (ECL) reagent was added and the samples were illuminated with X-rays, prior to scanning with a GDS8000 gel analysis system (UVP, LLC, Upland, CA, USA) to determine the density of specific light bands. The protein expression levels of each group were defined as the ratio between the density of light band in a given group and that of group S8.

#### Statistical analysis

Variables are expressed as the mean ± standard deviation. Differences between groups were analyzed with one way analysis of variance (ANOVA) using SPSS 16.0 (SPSS, Inc., Chicago, IL, USA). P<0.05 was considered to indicate a statistically significant difference.

## Results

### Effect of TSN on SBP

In all groups, the SBP began to rise in week 8 in the treatment and placebo groups. Compared with the baseline, SBP increased significantly by week 18 (P<0.01). However, a comparison of SBP between groups D18 and S18 did not reveal a significant difference (P>0.05). The comparison of SBP between groups is presented in Table I.

### Effect of TSN on LVH and myocardial fibrosis

LVMI, cardiac myocyte diameter and area, CVF and PVCA were significantly higher in group S18 compared with group S8 (as shown in Table I). All these indices were significantly lower in group D18 than in group S18.

### Histopathological study

**Figure 1:**
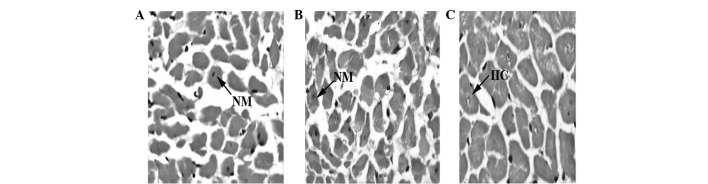
Effect of tanshinone IIA on myocardial cells in spontaneously hypertensive rats. Under hematoxylin and eosin staining (magnification, ×400), compared with groups (A) S8 and (B) D18, myocardial cells exhibited a greater extent of hypertrophy in (C) group S18. S8, control group; S18, placebo group; D18, treatment group; NM, normal myocyte; HC, hypertrophied cardiomyocyte.

**Figure 2:**
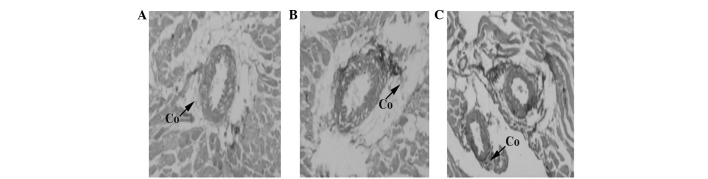
Effect of tanshinone IIA on collagen hyperplasia in spontaneously hypertensive rats. Under van Gieson staining (magnification, ×200), compared with groups (A) S8 and (B) D18, collagen hyperplasia was more prominent in (C) group S18. S8, control group; S18, placebo group; D18, treatment group; Co, collagen.

Under H&E and VG staining, SHRs in group S18 exhibited a greater extent of hypertrophic cardiac myocytes and collagen hyperplasia compared with those observed in group S8. Moreover, myocardial fibrosis and left ventricular hypertrophy were prominent in group S18. In contrast to group S18, SHRs treated with TSN in group D18 exhibited no significantly hypertrophic cardiac myocytes and collagen hyperplasia. The histopathological findings of groups S8, S18 and D18 are shown in Figs. 1 and 2.

### Cardiac myocyte apoptosis

Compared with group S8, cardiac apoptotic cell indices were higher in group S18. Following treatment with TSN, cardiac apoptotic cell indices decreased significantly compared with group S18. The comparison between groups is exhibited in Table II.

### Effect of TSN on myocardial tissue Bc1-2, Bax and p53 protein expression

**Figure 3:**
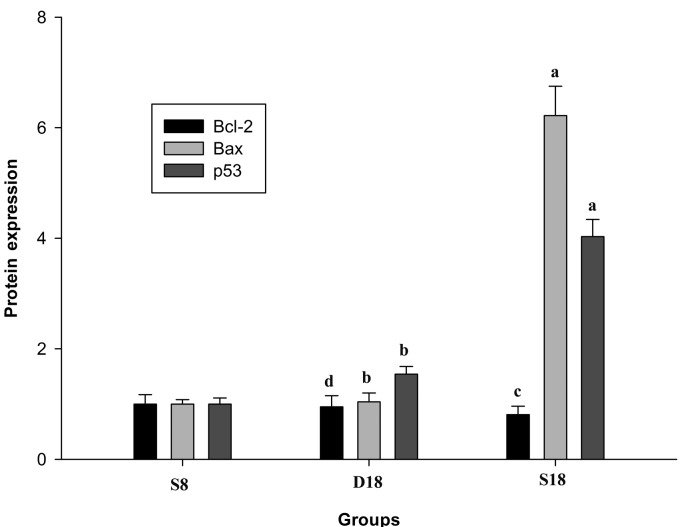
Effect of tanshinone IIA on myocardial tissue Bc1-2, Bax and p53 protein expression. Compared with group S18, protein expression levels of Bc1-2 were lower, while expression levels of Bax and p53 were higher, in groups S8 and D18. ^a^P<0.01 and ^c^P<0.05 compared with S8; ^b^P< 0.01 and ^d^P<0.05 compared with D18. S8, control group; S18, placebo group; D18, treatment group.

Rats in group S18 exhibited lower Bc1-2 protein expression levels (P<0.05 versus group S8) and higher p53 (P<0.01 versus group S8) and Bax (P<0.01 versus group S8) protein expression levels. Following treatment with TSN, Bc1-2 protein expression levels increased (P<0.05 versus group S18), while those of p53 (P<0.01 versus group S18) and Bax (P<0.01 versus group S18) decreased significantly. A comparison of protein expression levels between groups is shown in Table II and Fig. 3.

## Discussion

It has been identified that *Salvia miltiorrhiza* is rich in TSN, a member of the tanshinone family. Sodium tanshinone IIA sulfonate, extracted from *Salvia miltiorrhiza*, has been demonstrated to exert therapeutic effects in heart disease as a result of its vasodilative (4), atheroprotective (5), antiarrhythmic (6) and anti-inflammatory activities (7). Several studies have reported an improvement in clinical symptoms, signs and electrocardiography following the administration of TSN (11–14). Further research (15–18) has demonstrated that *Salvia miltiorrhiza* is able to block L-type calcium ion channels of ventricular myocardial cells, and therefore exerts calcium channel blocker-like effects. Moreover, inhibition of NF-κB expression in myocardial cells has also been reported (7). The present study demonstrated that *Salvia miltiorrhiza* attenuated LVH in SHRs, partly through the inhibition of RAAS activity. In a previous study (18), we focused on left ventricular norepinephrine (NE), epinephrine (E) and dopamine (DA) levels in SHRs. It was identified that NE and DA levels increased, while levels of E decreased in hypertrophic myocardial cells following administration of TSN. In addition to inhibiting the production of local cardiac angiotensin II, TSN also inhibited catecholamine release from sympathetic nerves, lowered the activation of α- and β-adrenergic receptors and, therefore, prevented SHRs from developing LVH. Furthermore, TSN downregulated the expression of cardiac protein kinase C to protect SHRs from LVH.

Apoptotic processes have been associated with LVH (10). In the present study, apoptotic processes were investigated in SHRs by observing the expression of Bcl-2, Bax and p53. It is well known that Bcl-2, also called the ‘antiapoptotic gene’, inhibits cell apoptosis, while Bax exerts a proapoptotic effect. Cell apoptosis depends on the homeostasis between Bax and Bcl-2 proteins. Inhibition of the apoptotic process has been shown to occur when Bcl-2 protein levels were higher than Bax protein levels, and vice versa (13). Gene p53 is known to upregulate Bax protein expression and downregulate Bcl-2 protein expression, and therefore exerts a proapoptotic effect (12–14). Results of the present study revealed that group S18 exhibited hypertrophic myocardial cells to a greater extent than groups S8 and D18. Under a state of LVH, Bax and p53 expression levels were significantly higher and Bcl-2 expression levels were lower than groups S8 and D18. The administration of TSN was demonstrated to be effective in upregulating Bcl-2 expression, while decreasing Bax and p53 expression. However, no significant difference was identified between the SBPs of groups S18 and D18. This indicated that the protective effect of TSN against LVH was not achieved via a decrease in blood pressure or an improvement in cardiac afterload. Through this study, we have shown that LVH is closely associated with apoptotic processes in SHRs, and that TSN is effective in preventing the development of LVH and downregulating apoptotic processes in SHRs. The exact mechanism by which apoptosis leads to LVH remains to be investigated.

In conclusion, the present study has demonstrated that LVH and apoptosis of cardiac tissues increase with the increasing age of SHRs. TSN may inhibit the development of LVH and decrease apoptotic processes in SHRs, possibly via upregulation of Bcl-2 and downregulation of Bax and p53 expression.

## Figures and Tables

**Table I:** Comparison of SBP, LVMI, CD, CA, CVF and PVCA between the control, placebo and treatment groups.

Group	SBP (mmHg)	LVMI (mg/g)	CD (μm)	CA (μm^2^)	CVF (%)	PVCA (%)
S8	147±9	2.86±0.22	16.26±2.1	218.43±52.44	3.77±0.57	2.46±0.38
D18	171±6^b^	3.23±0.24	16.35±1.84	230.23±69.37	4.54±0.80	2.94±0.56
S18	177±2^a^	4.28±0.68^a,c^	25.22±4.38^a,c^	490.12±118.96^a,c^	6.76±0.76^a,c^	4.86±0.65^a,c^

^a^P<0.01 and ^b^P<0.05 compared with S8; ^c^P<0.01 compared with D18. S8, control group; S18, placebo group; D18, treatment group; SBP, systolic blood pressure; LVMI, left ventricular mass index; CD, cardiac myocyte diameter; CA, cardiac myocyte area; CVF, collagen volume fraction; PVCA, perivascular circumferential area.

**Table II:** Comparison of cardiac apoptotic cells and protein expression levels in the groups of SHRs.

Group	Apoptotic cell index (%)	Bcl-2	Bax	p53
S8	9.45±1.81	1.00±0.17	1.00±0.08	1.00±0.11
D18	7.65±2.36	0.95±0.20	1.04±0.16	1.54±0.14
S18	11.59±1.48^a,b^	0.81±0.15^c,d^	6.22±0.53^a,b^	4.03±0.31^a,b^

^a^P<0.01 and ^c^P<0.05 compared with S8; ^b^P<0.01 and ^d^P<0.05 compared with D18. S8, control group; S18, placebo group; D18, treatment group; SHR, spontaneously hypertensive rat.
